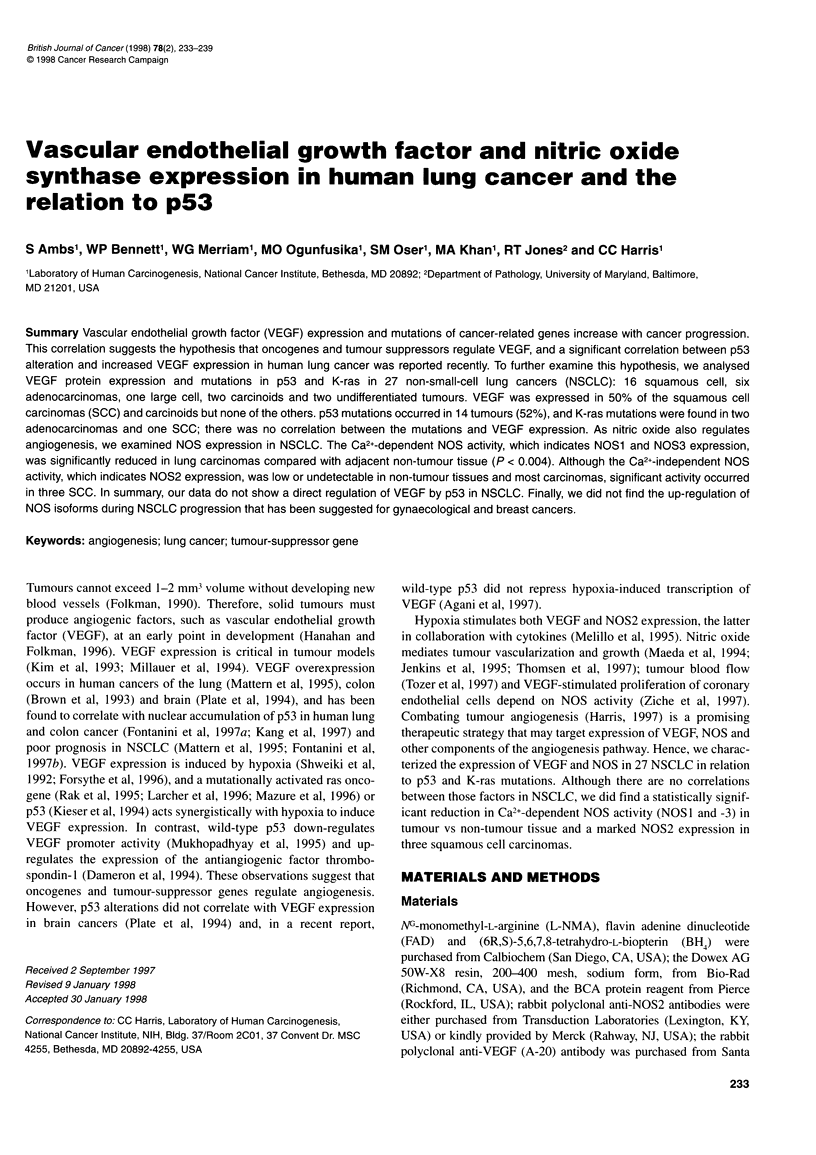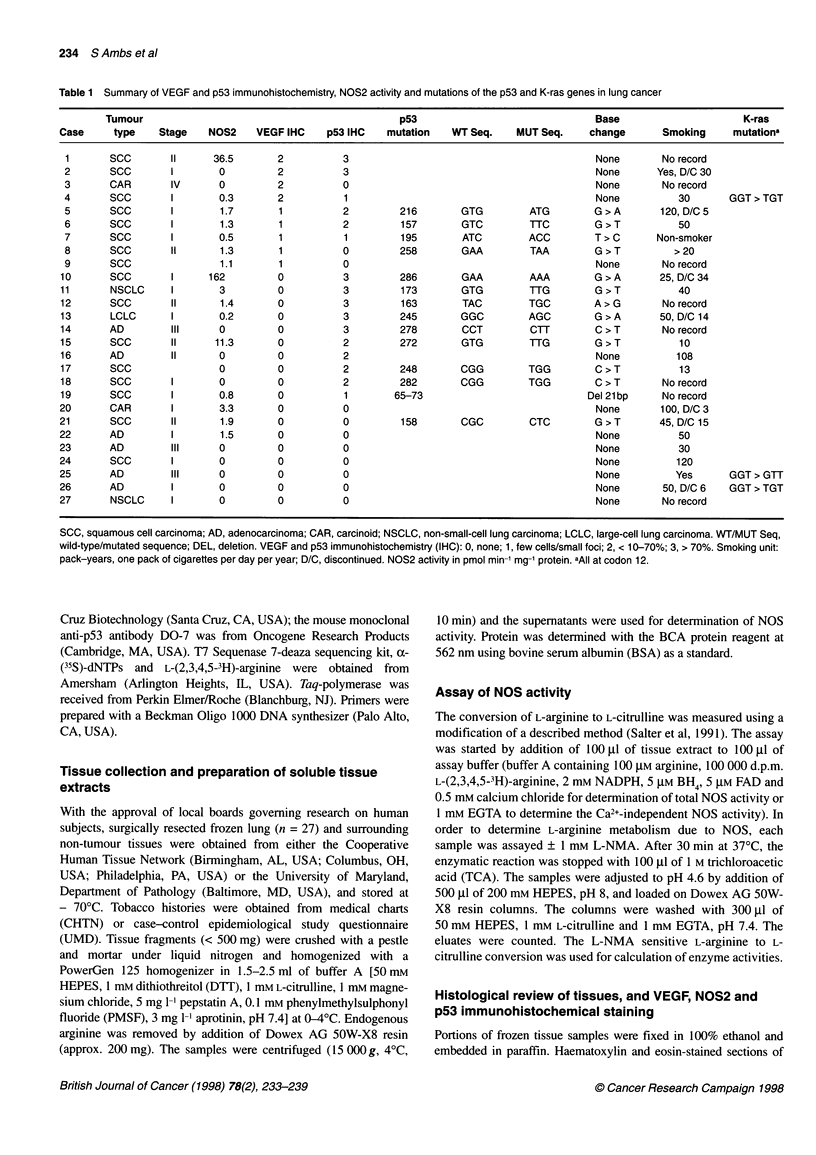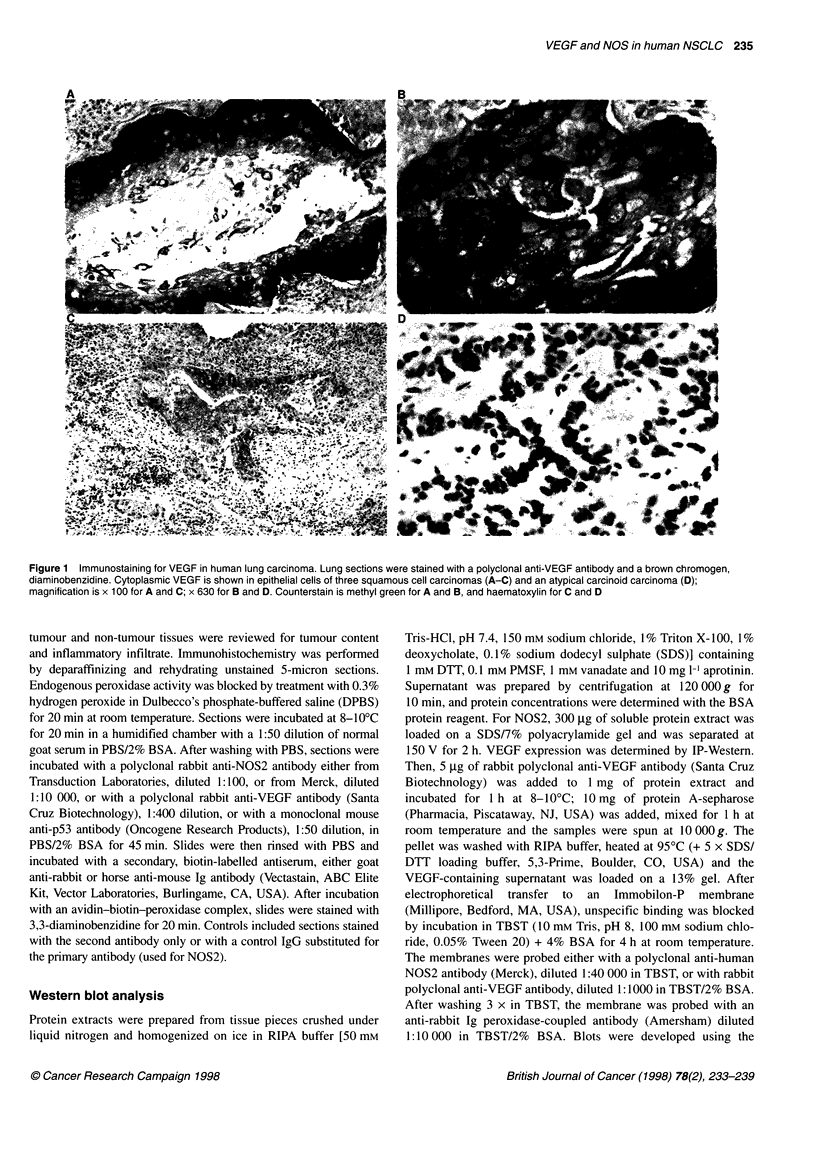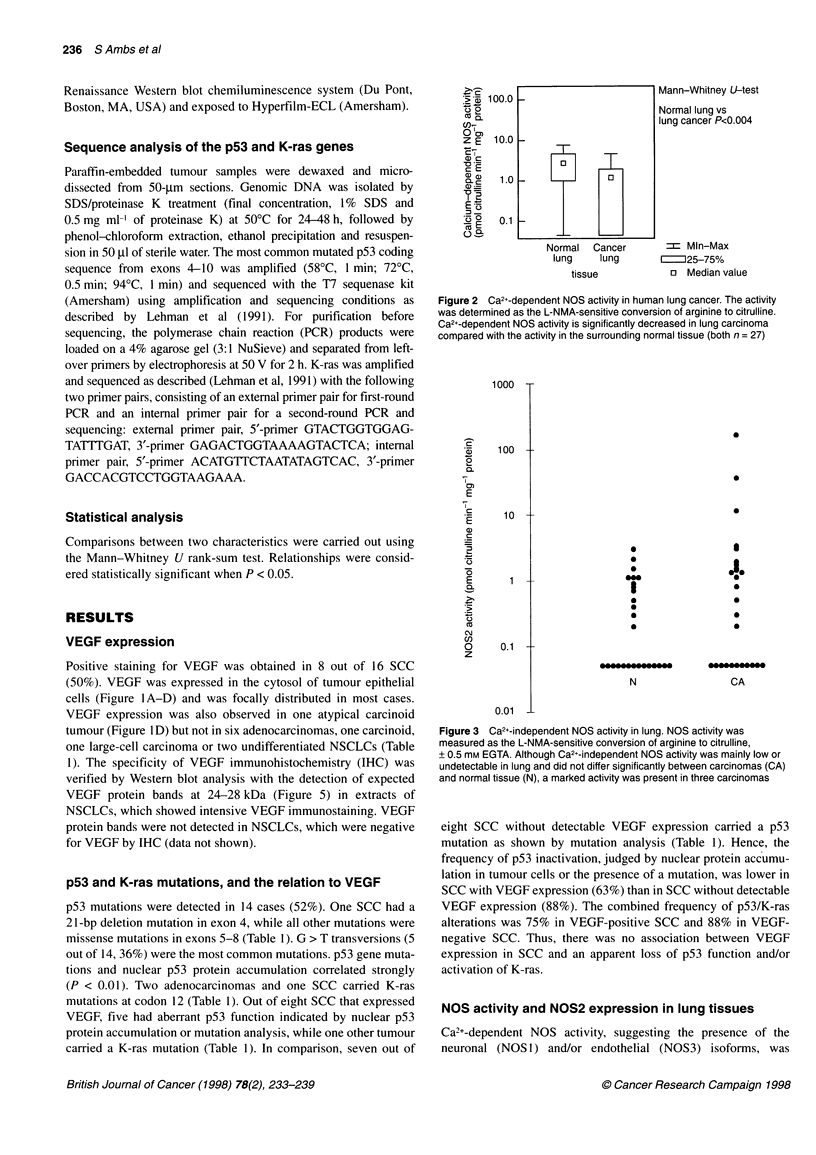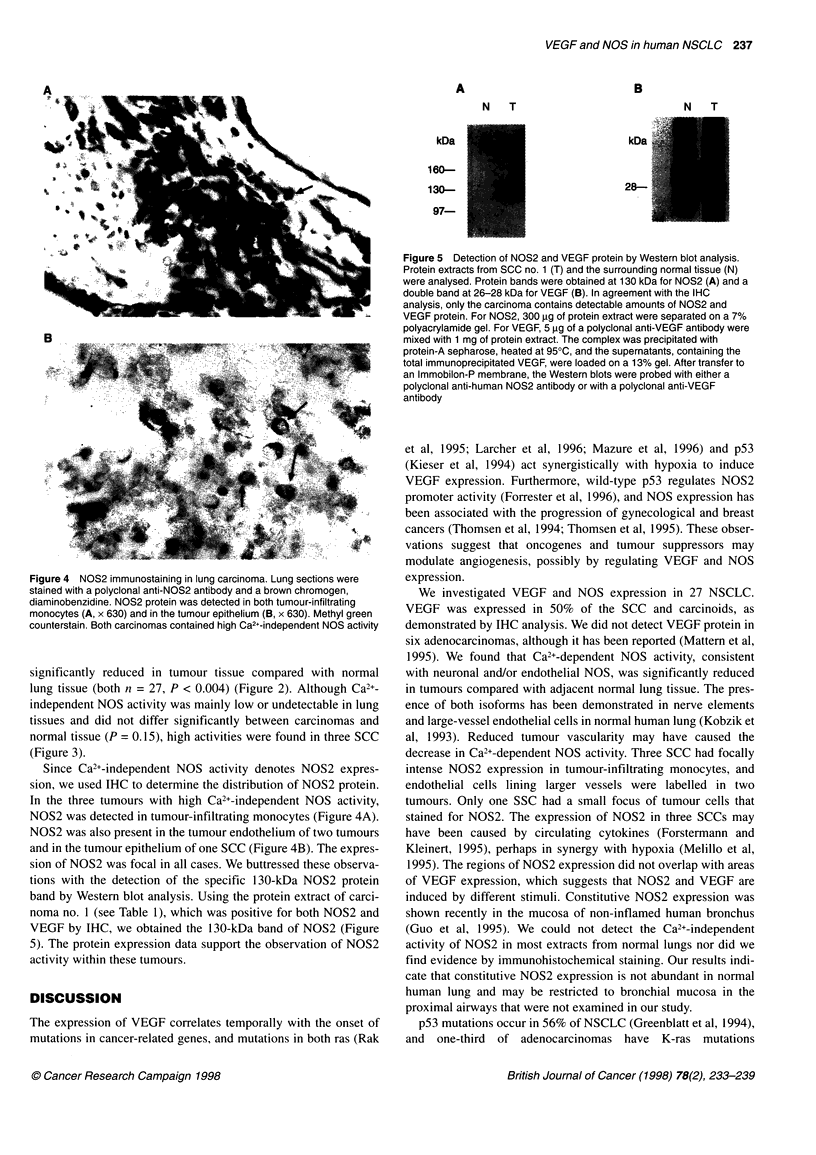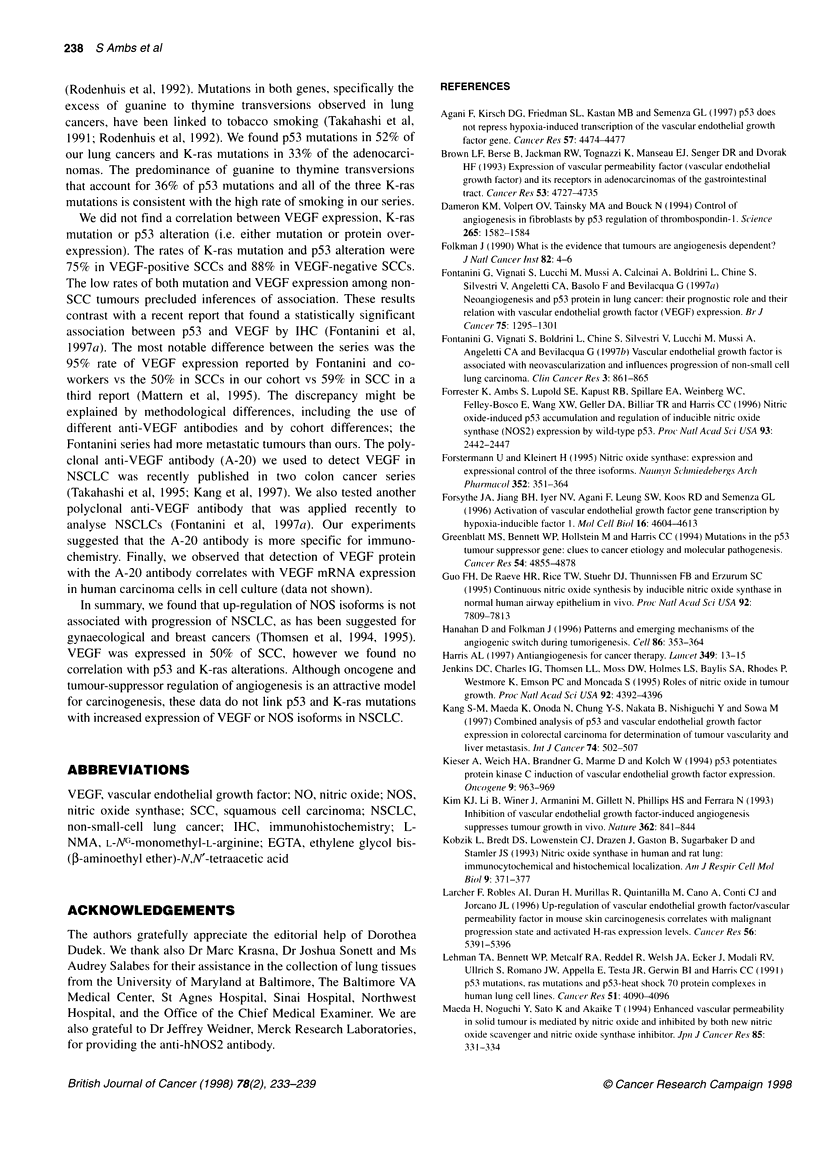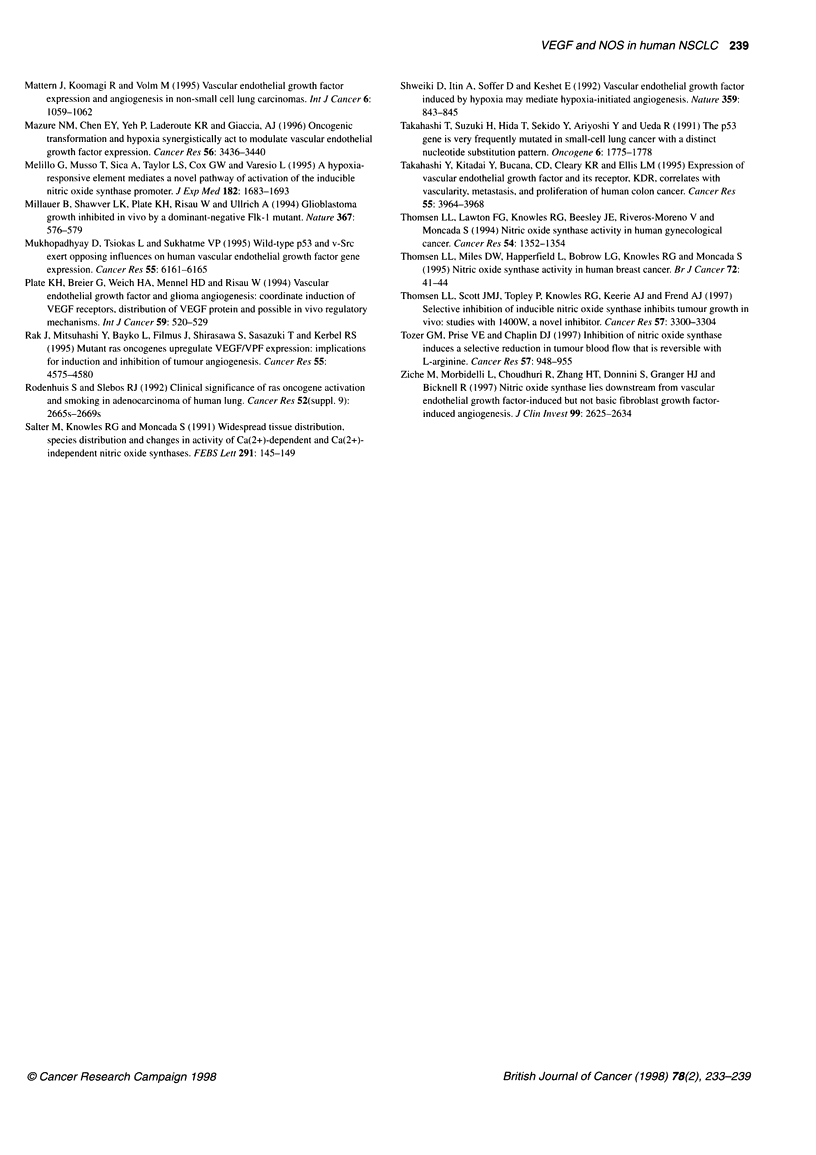# Vascular endothelial growth factor and nitric oxide synthase expression in human lung cancer and the relation to p53.

**DOI:** 10.1038/bjc.1998.470

**Published:** 1998-07

**Authors:** S. Ambs, W. P. Bennett, W. G. Merriam, M. O. Ogunfusika, S. M. Oser, M. A. Khan, R. T. Jones, C. C. Harris

**Affiliations:** Laboratory of Human Carcinogenesis, National Cancer Institute, Bethesda, MD 20892-4255, USA.

## Abstract

**Images:**


British Journal of Cancer (1998) 78(2), 233–239
© 1998 Cancer Research Campaign

### Vascular endothelial growth factor and nitric oxide synthase expression in human lung cancer and the relation to p53

S Ambs[1], WP Bennett[1], WG Merriam[1], MO Ogunfusika[1], SM Oser[1], MA Khan[1], RT Jones[2] and CC Harris[1]

[1]Laboratory of Human Carcinogenesis, National Cancer Institute, Bethesda, MD 20892; [2]Department of Pathology, University of Maryland, Baltimore, MD 21201, USA

**Summary** Vascular endothelial growth factor (VEGF) expression and mutations of cancer-related genes increase with cancer progression. This correlation suggests the hypothesis that oncogenes and tumour suppressors regulate VEGF, and a significant correlation between p53 alteration and increased VEGF expression in human lung cancer was reported recently. To further examine this hypothesis, we analysed VEGF protein expression and mutations in p53 and K-ras in 27 non-small-cell lung cancers (NSCLC): 16 squamous cell, six adenocarcinomas, one large cell, two carcinoids and two undifferentiated tumours. VEGF was expressed in 50% of the squamous cell carcinomas (SCC) and carcinoids but none of the others. p53 mutations occurred in 14 tumours (52%), and K-ras mutations were found in two adenocarcinomas and one SCC; there was no correlation between the mutations and VEGF expression. As nitric oxide also regulates angiogenesis, we examined NOS expression in NSCLC. The $Ca^{2+}$-dependent NOS activity, which indicates NOS1 and NOS3 expression, was significantly reduced in lung carcinomas compared with adjacent non-tumour tissue ($P < 0.004$). Although the $Ca^{2+}$-independent NOS activity, which indicates NOS2 expression, was low or undetectable in non-tumour tissues and most carcinomas, significant activity occurred in three SCC. In summary, our data do not show a direct regulation of VEGF by p53 in NSCLC. Finally, we did not find the up-regulation of NOS isoforms during NSCLC progression that has been suggested for gynaecological and breast cancers.

**Keywords:** angiogenesis; lung cancer; tumour-suppressor gene

Tumours cannot exceed 1–2 $mm^3$ volume without developing new blood vessels (Folkman, 1990). Therefore, solid tumours must produce angiogenic factors, such as vascular endothelial growth factor (VEGF), at an early point in development (Hanahan and Folkman, 1996). VEGF expression is critical in tumour models (Kim et al, 1993; Millauer et al, 1994). VEGF overexpression occurs in human cancers of the lung (Mattern et al, 1995), colon (Brown et al, 1993) and brain (Plate et al, 1994), and has been found to correlate with nuclear accumulation of p53 in human lung and colon cancer (Fontanini et al, 1997*a*; Kang et al, 1997) and poor prognosis in NSCLC (Mattern et al, 1995; Fontanini et al, 1997*b*). VEGF expression is induced by hypoxia (Shweiki et al, 1992; Forsythe et al, 1996), and a mutationally activated ras oncogene (Rak et al, 1995; Larcher et al, 1996; Mazure et al, 1996) or p53 (Kieser et al, 1994) acts synergistically with hypoxia to induce VEGF expression. In contrast, wild-type p53 down-regulates VEGF promoter activity (Mukhopadhyay et al, 1995) and up-regulates the expression of the antiangiogenic factor thrombospondin-1 (Dameron et al, 1994). These observations suggest that oncogenes and tumour-suppressor genes regulate angiogenesis. However, p53 alterations did not correlate with VEGF expression in brain cancers (Plate et al, 1994) and, in a recent report, wild-type p53 did not repress hypoxia-induced transcription of VEGF (Agani et al, 1997).

Hypoxia stimulates both VEGF and NOS2 expression, the latter in collaboration with cytokines (Melillo et al, 1995). Nitric oxide mediates tumour vascularization and growth (Maeda et al, 1994; Jenkins et al, 1995; Thomsen et al, 1997); tumour blood flow (Tozer et al, 1997) and VEGF-stimulated proliferation of coronary endothelial cells depend on NOS activity (Ziche et al, 1997). Combating tumour angiogenesis (Harris, 1997) is a promising therapeutic strategy that may target expression of VEGF, NOS and other components of the angiogenesis pathway. Hence, we characterized the expression of VEGF and NOS in 27 NSCLC in relation to p53 and K-ras mutations. Although there are no correlations between those factors in NSCLC, we did find a statistically significant reduction in $Ca^{2+}$-dependent NOS activity (NOS1 and -3) in tumour vs non-tumour tissue and a marked NOS2 expression in three squamous cell carcinomas.

### MATERIALS AND METHODS

### Materials

$N^G$-monomethyl-L-arginine (L-NMA), flavin adenine dinucleotide (FAD) and (6R,S)-5,6,7,8-tetrahydro-L-biopterin ($BH_4$) were purchased from Calbiochem (San Diego, CA, USA); the Dowex AG 50W-X8 resin, 200–400 mesh, sodium form, from Bio-Rad (Richmond, CA, USA), and the BCA protein reagent from Pierce (Rockford, IL, USA); rabbit polyclonal anti-NOS2 antibodies were either purchased from Transduction Laboratories (Lexington, KY, USA) or kindly provided by Merck (Rahway, NJ, USA); the rabbit polyclonal anti-VEGF (A-20) antibody was purchased from Santa

*Received 2 September 1997*
*Revised 9 January 1998*
*Accepted 30 January 1998*

*Correspondence to:* CC Harris, Laboratory of Human Carcinogenesis, National Cancer Institute, NIH, Bldg. 37/Room 2C01, 37 Convent Dr. MSC 4255, Bethesda, MD 20892-4255, USA

**Table 1** Summary of VEGF and p53 immunohistochemistry, NOS2 activity and mutations of the p53 and K-ras genes in lung cancer

| Case | Tumour type | Stage | NOS2 | VEGF IHC | p53 IHC | p53 mutation | WT Seq. | MUT Seq. | Base change | Smoking | K-ras mutation[a] |
|---|---|---|---|---|---|---|---|---|---|---|---|
| 1 | SCC | II | 36.5 | 2 | 3 | | | | None | No record | |
| 2 | SCC | I | 0 | 2 | 3 | | | | None | Yes, D/C 30 | |
| 3 | CAR | IV | 0 | 2 | 0 | | | | None | No record | |
| 4 | SCC | I | 0.3 | 2 | 1 | | | | None | 30 | GGT > TGT |
| 5 | SCC | I | 1.7 | 1 | 2 | 216 | GTG | ATG | G > A | 120, D/C 5 | |
| 6 | SCC | I | 1.3 | 1 | 2 | 157 | GTC | TTC | G > T | 50 | |
| 7 | SCC | I | 0.5 | 1 | 1 | 195 | ATC | ACC | T > C | Non-smoker | |
| 8 | SCC | II | 1.3 | 1 | 0 | 258 | GAA | TAA | G > T | > 20 | |
| 9 | SCC | | 1.1 | 1 | 0 | | | | None | No record | |
| 10 | SCC | I | 162 | 0 | 3 | 286 | GAA | AAA | G > A | 25, D/C 34 | |
| 11 | NSCLC | I | 3 | 0 | 3 | 173 | GTG | TTG | G > T | 40 | |
| 12 | SCC | II | 1.4 | 0 | 3 | 163 | TAC | TGC | A > G | No record | |
| 13 | LCLC | I | 0.2 | 0 | 3 | 245 | GGC | AGC | G > A | 50, D/C 14 | |
| 14 | AD | III | 0 | 0 | 3 | 278 | CCT | CTT | C > T | No record | |
| 15 | SCC | II | 11.3 | 0 | 2 | 272 | GTG | TTG | G > T | 10 | |
| 16 | AD | II | 0 | 0 | 2 | | | | None | 108 | |
| 17 | SCC | | 0 | 0 | 2 | 248 | CGG | TGG | C > T | 13 | |
| 18 | SCC | I | 0 | 0 | 2 | 282 | CGG | TGG | C > T | No record | |
| 19 | SCC | I | 0.8 | 0 | 1 | 65–73 | | | Del 21bp | No record | |
| 20 | CAR | I | 3.3 | 0 | 0 | | | | None | 100, D/C 3 | |
| 21 | SCC | II | 1.9 | 0 | 0 | 158 | CGC | CTC | G > T | 45, D/C 15 | |
| 22 | AD | I | 1.5 | 0 | 0 | | | | None | 50 | |
| 23 | AD | III | 0 | 0 | 0 | | | | None | 30 | |
| 24 | SCC | I | 0 | 0 | 0 | | | | None | 120 | |
| 25 | AD | III | 0 | 0 | 0 | | | | None | Yes | GGT > GTT |
| 26 | AD | I | 0 | 0 | 0 | | | | None | 50, D/C 6 | GGT > TGT |
| 27 | NSCLC | I | 0 | 0 | 0 | | | | None | No record | |

SCC, squamous cell carcinoma; AD, adenocarcinoma; CAR, carcinoid; NSCLC, non-small-cell lung carcinoma; LCLC, large-cell lung carcinoma. WT/MUT Seq, wild-type/mutated sequence; DEL, deletion. VEGF and p53 immunohistochemistry (IHC): 0, none; 1, few cells/small foci; 2, < 10–70%; 3, > 70%. Smoking unit: pack–years, one pack of cigarettes per day per year; D/C, discontinued. NOS2 activity in pmol $min^{-1}$ $mg^{-1}$ protein. [a]All at codon 12.

Cruz Biotechnology (Santa Cruz, CA, USA); the mouse monoclonal anti-p53 antibody DO-7 was from Oncogene Research Products (Cambridge, MA, USA). T7 Sequenase 7-deaza sequencing kit, α-($^{35}$S)-dNTPs and L-(2,3,4,5-$^{3}$H)-arginine were obtained from Amersham (Arlington Heights, IL, USA). *Taq*-polymerase was received from Perkin Elmer/Roche (Blanchburg, NJ). Primers were prepared with a Beckman Oligo 1000 DNA synthesizer (Palo Alto, CA, USA).

### Tissue collection and preparation of soluble tissue extracts

With the approval of local boards governing research on human subjects, surgically resected frozen lung ($n$ = 27) and surrounding non-tumour tissues were obtained from either the Cooperative Human Tissue Network (Birmingham, AL, USA; Columbus, OH, USA; Philadelphia, PA, USA) or the University of Maryland, Department of Pathology (Baltimore, MD, USA), and stored at − 70°C. Tobacco histories were obtained from medical charts (CHTN) or case–control epidemiological study questionnaire (UMD). Tissue fragments (< 500 mg) were crushed with a pestle and mortar under liquid nitrogen and homogenized with a PowerGen 125 homogenizer in 1.5–2.5 ml of buffer A [50 mM HEPES, 1 mM dithiothreitol (DTT), 1 mM L-citrulline, 1 mM magnesium chloride, 5 mg $l^{-1}$ pepstatin A, 0.1 mM phenylmethylsulphonyl fluoride (PMSF), 3 mg $l^{-1}$ aprotinin, pH 7.4] at 0–4°C. Endogenous arginine was removed by addition of Dowex AG 50W-X8 resin (approx. 200 mg). The samples were centrifuged (15 000 ***g***, 4°C, 10 min) and the supernatants were used for determination of NOS activity. Protein was determined with the BCA protein reagent at 562 nm using bovine serum albumin (BSA) as a standard.

### Assay of NOS activity

The conversion of L-arginine to L-citrulline was measured using a modification of a described method (Salter et al, 1991). The assay was started by addition of 100 μl of tissue extract to 100 μl of assay buffer (buffer A containing 100 μM arginine, 100 000 d.p.m. L-(2,3,4,5-$^{3}$H)-arginine, 2 mM NADPH, 5 μM $BH_4$, 5 μM FAD and 0.5 mM calcium chloride for determination of total NOS activity or 1 mM EGTA to determine the $Ca^{2+}$-independent NOS activity). In order to determine L-arginine metabolism due to NOS, each sample was assayed ± 1 mM L-NMA. After 30 min at 37°C, the enzymatic reaction was stopped with 100 μl of 1 M trichloroacetic acid (TCA). The samples were adjusted to pH 4.6 by addition of 500 μl of 200 mM HEPES, pH 8, and loaded on Dowex AG 50W-X8 resin columns. The columns were washed with 300 μl of 50 mM HEPES, 1 mM L-citrulline and 1 mM EGTA, pH 7.4. The eluates were counted. The L-NMA sensitive L-arginine to L-citrulline conversion was used for calculation of enzyme activities.

### Histological review of tissues, and VEGF, NOS2 and p53 immunohistochemical staining

Portions of frozen tissue samples were fixed in 100% ethanol and embedded in paraffin. Haematoxylin and eosin-stained sections of

© Cancer Research Campaign 1998

**Figure 1** Immunostaining for VEGF in human lung carcinoma. Lung sections were stained with a polyclonal anti-VEGF antibody and a brown chromogen, diaminobenzidine. Cytoplasmic VEGF is shown in epithelial cells of three squamous cell carcinomas (**A–C**) and an atypical carcinoid carcinoma (**D**); magnification is × 100 for **A** and **C**; × 630 for **B** and **D**. Counterstain is methyl green for **A** and **B**, and haematoxylin for **C** and **D**

tumour and non-tumour tissues were reviewed for tumour content and inflammatory infiltrate. Immunohistochemistry was performed by deparaffinizing and rehydrating unstained 5-micron sections. Endogenous peroxidase activity was blocked by treatment with 0.3% hydrogen peroxide in Dulbecco's phosphate-buffered saline (DPBS) for 20 min at room temperature. Sections were incubated at 8–10°C for 20 min in a humidified chamber with a 1:50 dilution of normal goat serum in PBS/2% BSA. After washing with PBS, sections were incubated with a polyclonal rabbit anti-NOS2 antibody either from Transduction Laboratories, diluted 1:100, or from Merck, diluted 1:10 000, or with a polyclonal rabbit anti-VEGF antibody (Santa Cruz Biotechnology), 1:400 dilution, or with a monoclonal mouse anti-p53 antibody (Oncogene Research Products), 1:50 dilution, in PBS/2% BSA for 45 min. Slides were then rinsed with PBS and incubated with a secondary, biotin-labelled antiserum, either goat anti-rabbit or horse anti-mouse Ig antibody (Vectastain, ABC Elite Kit, Vector Laboratories, Burlingame, CA, USA). After incubation with an avidin–biotin–peroxidase complex, slides were stained with 3,3-diaminobenzidine for 20 min. Controls included sections stained with the second antibody only or with a control IgG substituted for the primary antibody (used for NOS2).

### Western blot analysis

Protein extracts were prepared from tissue pieces crushed under liquid nitrogen and homogenized on ice in RIPA buffer [50 mM Tris-HCl, pH 7.4, 150 mM sodium chloride, 1% Triton X-100, 1% deoxycholate, 0.1% sodium dodecyl sulphate (SDS)] containing 1 mM DTT, 0.1 mM PMSF, 1 mM vanadate and 10 mg $l^{-1}$ aprotinin. Supernatant was prepared by centrifugation at 120 000 ***g*** for 10 min, and protein concentrations were determined with the BSA protein reagent. For NOS2, 300 μg of soluble protein extract was loaded on a SDS/7% polyacrylamide gel and was separated at 150 V for 2 h. VEGF expression was determined by IP-Western. Then, 5 μg of rabbit polyclonal anti-VEGF antibody (Santa Cruz Biotechnology) was added to 1 mg of protein extract and incubated for 1 h at 8–10°C; 10 mg of protein A-sepharose (Pharmacia, Piscataway, NJ, USA) was added, mixed for 1 h at room temperature and the samples were spun at 10 000 ***g***. The pellet was washed with RIPA buffer, heated at 95°C (+ 5 × SDS/DTT loading buffer, 5,3-Prime, Boulder, CO, USA) and the VEGF-containing supernatant was loaded on a 13% gel. After electrophoretical transfer to an Immobilon-P membrane (Millipore, Bedford, MA, USA), unspecific binding was blocked by incubation in TBST (10 mM Tris, pH 8, 100 mM sodium chloride, 0.05% Tween 20) + 4% BSA for 4 h at room temperature. The membranes were probed either with a polyclonal anti-human NOS2 antibody (Merck), diluted 1:40 000 in TBST, or with rabbit polyclonal anti-VEGF antibody, diluted 1:1000 in TBST/2% BSA. After washing 3 × in TBST, the membrane was probed with an anti-rabbit Ig peroxidase-coupled antibody (Amersham) diluted 1:10 000 in TBST/2% BSA. Blots were developed using the

© Cancer Research Campaign 1998

Renaissance Western blot chemiluminescence system (Du Pont, Boston, MA, USA) and exposed to Hyperfilm-ECL (Amersham).

### Sequence analysis of the p53 and K-ras genes

Paraffin-embedded tumour samples were dewaxed and microdissected from 50-µm sections. Genomic DNA was isolated by SDS/proteinase K treatment (final concentration, 1% SDS and 0.5 mg $ml^{-1}$ of proteinase K) at 50°C for 24–48 h, followed by phenol–chloroform extraction, ethanol precipitation and resuspension in 50 µl of sterile water. The most common mutated p53 coding sequence from exons 4–10 was amplified (58°C, 1 min; 72°C, 0.5 min; 94°C, 1 min) and sequenced with the T7 sequenase kit (Amersham) using amplification and sequencing conditions as described by Lehman et al (1991). For purification before sequencing, the polymerase chain reaction (PCR) products were loaded on a 4% agarose gel (3:1 NuSieve) and separated from left-over primers by electrophoresis at 50 V for 2 h. K-ras was amplified and sequenced as described (Lehman et al, 1991) with the following two primer pairs, consisting of an external primer pair for first-round PCR and an internal primer pair for a second-round PCR and sequencing: external primer pair, 5′-primer GTACTGGTGGAG-TATTTGAT, 3′-primer GAGACTGGTAAAAGTACTCA; internal primer pair, 5′-primer ACATGTTCTAATATAGTCAC, 3′-primer GACCACGTCCTGGTAAGAAA.

### Statistical analysis

Comparisons between two characteristics were carried out using the Mann–Whitney *U* rank-sum test. Relationships were considered statistically significant when $P < 0.05$.

### RESULTS

### VEGF expression

Positive staining for VEGF was obtained in 8 out of 16 SCC (50%). VEGF was expressed in the cytosol of tumour epithelial cells (Figure 1A–D) and was focally distributed in most cases. VEGF expression was also observed in one atypical carcinoid tumour (Figure 1D) but not in six adenocarcinomas, one carcinoid, one large-cell carcinoma or two undifferentiated NSCLCs (Table 1). The specificity of VEGF immunohistochemistry (IHC) was verified by Western blot analysis with the detection of expected VEGF protein bands at 24–28 kDa (Figure 5) in extracts of NSCLCs, which showed intensive VEGF immunostaining. VEGF protein bands were not detected in NSCLCs, which were negative for VEGF by IHC (data not shown).

### p53 and K-ras mutations, and the relation to VEGF

p53 mutations were detected in 14 cases (52%). One SCC had a 21-bp deletion mutation in exon 4, while all other mutations were missense mutations in exons 5–8 (Table 1). G > T transversions (5 out of 14, 36%) were the most common mutations. p53 gene mutations and nuclear p53 protein accumulation correlated strongly ($P < 0.01$). Two adenocarcinomas and one SCC carried K-ras mutations at codon 12 (Table 1). Out of eight SCC that expressed VEGF, five had aberrant p53 function indicated by nuclear p53 protein accumulation or mutation analysis, while one other tumour carried a K-ras mutation (Table 1). In comparison, seven out of

**Figure 2** $Ca^{2+}$-dependent NOS activity in human lung cancer. The activity was determined as the L-NMA-sensitive conversion of arginine to citrulline. $Ca^{2+}$-dependent NOS activity is significantly decreased in lung carcinoma compared with the activity in the surrounding normal tissue (both $n = 27$)

**Figure 3** $Ca^{2+}$-independent NOS activity in lung. NOS activity was measured as the L-NMA-sensitive conversion of arginine to citrulline, ± 0.5 mM EGTA. Although $Ca^{2+}$-independent NOS activity was mainly low or undetectable in lung and did not differ significantly between carcinomas (CA) and normal tissue (N), a marked activity was present in three carcinomas eight SCC without detectable VEGF expression carried a p53 mutation as shown by mutation analysis (Table 1). Hence, the frequency of p53 inactivation, judged by nuclear protein accumulation in tumour cells or the presence of a mutation, was lower in SCC with VEGF expression (63%) than in SCC without detectable VEGF expression (88%). The combined frequency of p53/K-ras alterations was 75% in VEGF-positive SCC and 88% in VEGF-negative SCC. Thus, there was no association between VEGF expression in SCC and an apparent loss of p53 function and/or activation of K-ras.

### NOS activity and NOS2 expression in lung tissues

$Ca^{2+}$-dependent NOS activity, suggesting the presence of the neuronal (NOS1) and/or endothelial (NOS3) isoforms, was

© Cancer Research Campaign 1998

Figure 4 NOS2 immunostaining in lung carcinoma. Lung sections were stained with a polyclonal anti-NOS2 antibody and a brown chromogen, diaminobenzidine. NOS2 protein was detected in both tumour-infiltrating monocytes (**A**, × 630) and in the tumour epithelium (**B**, × 630). Methyl green counterstain. Both carcinomas contained high $Ca^{2+}$-independent NOS activity

**Figure 5** Detection of NOS2 and VEGF protein by Western blot analysis. Protein extracts from SCC no. 1 (T) and the surrounding normal tissue (N) were analysed. Protein bands were obtained at 130 kDa for NOS2 (**A**) and a double band at 26–28 kDa for VEGF (**B**). In agreement with the IHC analysis, only the carcinoma contains detectable amounts of NOS2 and VEGF protein. For NOS2, 300 μg of protein extract were separated on a 7% polyacrylamide gel. For VEGF, 5 μg of a polyclonal anti-VEGF antibody were mixed with 1 mg of protein extract. The complex was precipitated with protein-A sepharose, heated at 95°C, and the supernatants, containing the total immunoprecipitated VEGF, were loaded on a 13% gel. After transfer to an Immobilon-P membrane, the Western blots were probed with either a polyclonal anti-human NOS2 antibody or with a polyclonal anti-VEGF antibody significantly reduced in tumour tissue compared with normal lung tissue (both $n = 27$, $P < 0.004$) (Figure 2). Although $Ca^{2+}$-independent NOS activity was mainly low or undetectable in lung tissues and did not differ significantly between carcinomas and normal tissue ($P = 0.15$), high activities were found in three SCC (Figure 3).

Since $Ca^{2+}$-independent NOS activity denotes NOS2 expression, we used IHC to determine the distribution of NOS2 protein. In the three tumours with high $Ca^{2+}$-independent NOS activity, NOS2 was detected in tumour-infiltrating monocytes (Figure 4A). NOS2 was also present in the tumour endothelium of two tumours and in the tumour epithelium of one SCC (Figure 4B). The expression of NOS2 was focal in all cases. We buttressed these observations with the detection of the specific 130-kDa NOS2 protein band by Western blot analysis. Using the protein extract of carcinoma no. 1 (see Table 1), which was positive for both NOS2 and VEGF by IHC, we obtained the 130-kDa band of NOS2 (Figure 5). The protein expression data support the observation of NOS2 activity within these tumours.

### DISCUSSION

The expression of VEGF correlates temporally with the onset of mutations in cancer-related genes, and mutations in both ras (Rak et al, 1995; Larcher et al, 1996; Mazure et al, 1996) and p53 (Kieser et al, 1994) act synergistically with hypoxia to induce VEGF expression. Furthermore, wild-type p53 regulates NOS2 promoter activity (Forrester et al, 1996), and NOS expression has been associated with the progression of gynecological and breast cancers (Thomsen et al, 1994; Thomsen et al, 1995). These observations suggest that oncogenes and tumour suppressors may modulate angiogenesis, possibly by regulating VEGF and NOS expression.

We investigated VEGF and NOS expression in 27 NSCLC. VEGF was expressed in 50% of the SCC and carcinoids, as demonstrated by IHC analysis. We did not detect VEGF protein in six adenocarcinomas, although it has been reported (Mattern et al, 1995). We found that $Ca^{2+}$-dependent NOS activity, consistent with neuronal and/or endothelial NOS, was significantly reduced in tumours compared with adjacent normal lung tissue. The presence of both isoforms has been demonstrated in nerve elements and large-vessel endothelial cells in normal human lung (Kobzik et al, 1993). Reduced tumour vascularity may have caused the decrease in $Ca^{2+}$-dependent NOS activity. Three SCC had focally intense NOS2 expression in tumour-infiltrating monocytes, and endothelial cells lining larger vessels were labelled in two tumours. Only one SSC had a small focus of tumour cells that stained for NOS2. The expression of NOS2 in three SCCs may have been caused by circulating cytokines (Forstermann and Kleinert, 1995), perhaps in synergy with hypoxia (Melillo et al, 1995). The regions of NOS2 expression did not overlap with areas of VEGF expression, which suggests that NOS2 and VEGF are induced by different stimuli. Constitutive NOS2 expression was shown recently in the mucosa of non-inflamed human bronchus (Guo et al, 1995). We could not detect the $Ca^{2+}$-independent activity of NOS2 in most extracts from normal lungs nor did we find evidence by immunohistochemical staining. Our results indicate that constitutive NOS2 expression is not abundant in normal human lung and may be restricted to bronchial mucosa in the proximal airways that were not examined in our study.

p53 mutations occur in 56% of NSCLC (Greenblatt et al, 1994), and one-third of adenocarcinomas have K-ras mutations

© Cancer Research Campaign 1998 

(Rodenhuis et al, 1992). Mutations in both genes, specifically the excess of guanine to thymine transversions observed in lung cancers, have been linked to tobacco smoking (Takahashi et al, 1991; Rodenhuis et al, 1992). We found p53 mutations in 52% of our lung cancers and K-ras mutations in 33% of the adenocarcinomas. The predominance of guanine to thymine transversions that account for 36% of p53 mutations and all of the three K-ras mutations is consistent with the high rate of smoking in our series.

We did not find a correlation between VEGF expression, K-ras mutation or p53 alteration (i.e. either mutation or protein overexpression). The rates of K-ras mutation and p53 alteration were 75% in VEGF-positive SCCs and 88% in VEGF-negative SCCs. The low rates of both mutation and VEGF expression among non-SCC tumours precluded inferences of association. These results contrast with a recent report that found a statistically significant association between p53 and VEGF by IHC (Fontanini et al, 1997*a*). The most notable difference between the series was the 95% rate of VEGF expression reported by Fontanini and coworkers vs the 50% in SCCs in our cohort vs 59% in SCC in a third report (Mattern et al, 1995). The discrepancy might be explained by methodological differences, including the use of different anti-VEGF antibodies and by cohort differences; the Fontanini series had more metastatic tumours than ours. The polyclonal anti-VEGF antibody (A-20) we used to detect VEGF in NSCLC was recently published in two colon cancer series (Takahashi et al, 1995; Kang et al, 1997). We also tested another polyclonal anti-VEGF antibody that was applied recently to analyse NSCLCs (Fontanini et al, 1997*a*). Our experiments suggested that the A-20 antibody is more specific for immunochemistry. Finally, we observed that detection of VEGF protein with the A-20 antibody correlates with VEGF mRNA expression in human carcinoma cells in cell culture (data not shown).

In summary, we found that up-regulation of NOS isoforms is not associated with progression of NSCLC, as has been suggested for gynaecological and breast cancers (Thomsen et al, 1994, 1995). VEGF was expressed in 50% of SCC, however we found no correlation with p53 and K-ras alterations. Although oncogene and tumour-suppressor regulation of angiogenesis is an attractive model for carcinogenesis, these data do not link p53 and K-ras mutations with increased expression of VEGF or NOS isoforms in NSCLC.

### ABBREVIATIONS

VEGF, vascular endothelial growth factor; NO, nitric oxide; NOS, nitric oxide synthase; SCC, squamous cell carcinoma; NSCLC, non-small-cell lung cancer; IHC, immunohistochemistry; L-NMA, L-$N^G$-monomethyl-L-arginine; EGTA, ethylene glycol bis-(β-aminoethyl ether)-*N*,*N'*-tetraacetic acid

### ACKNOWLEDGEMENTS

The authors gratefully appreciate the editorial help of Dorothea Dudek. We thank also Dr Marc Krasna, Dr Joshua Sonett and Ms Audrey Salabes for their assistance in the collection of lung tissues from the University of Maryland at Baltimore, The Baltimore VA Medical Center, St Agnes Hospital, Sinai Hospital, Northwest Hospital, and the Office of the Chief Medical Examiner. We are also grateful to Dr Jeffrey Weidner, Merck Research Laboratories, for providing the anti-hNOS2 antibody.

### REFERENCES

Agani F, Kirsch DG, Friedman SL, Kastan MB and Semenza GL (1997) p53 does not repress hypoxia-induced transcription of the vascular endothelial growth factor gene. *Cancer Res* **57**: 4474–4477

Brown LF, Berse B, Jackman RW, Tognazzi K, Manseau EJ, Senger DR and Dvorak HF (1993) Expression of vascular permeability factor (vascular endothelial growth factor) and its receptors in adenocarcinomas of the gastrointestinal tract. *Cancer Res* **53**: 4727–4735

Dameron KM, Volpert OV, Tainsky MA and Bouck N (1994) Control of angiogenesis in fibroblasts by p53 regulation of thrombospondin-1. *Science* **265**: 1582–1584

Folkman J (1990) What is the evidence that tumours are angiogenesis dependent? *J Natl Cancer Inst* **82**: 4–6

Fontanini G, Vignati S, Lucchi M, Mussi A, Calcinai A, Boldrini L, Chine S, Silvestri V, Angeletti CA, Basolo F and Bevilacqua G (1997*a*) Neoangiogenesis and p53 protein in lung cancer: their prognostic role and their relation with vascular endothelial growth factor (VEGF) expression. *Br J Cancer* **75**: 1295–1301

Fontanini G, Vignati S, Boldrini L, Chine S, Silvestri V, Lucchi M, Mussi A, Angeletti CA and Bevilacqua G (1997*b*) Vascular endothelial growth factor is associated with neovascularization and influences progression of non-small cell lung carcinoma. *Clin Cancer Res* **3**: 861–865

Forrester K, Ambs S, Lupold SE, Kapust RB, Spillare EA, Weinberg WC, Felley-Bosco E, Wang XW, Geller DA, Billiar TR and Harris CC (1996) Nitric oxide-induced p53 accumulation and regulation of inducible nitric oxide synthase (NOS2) expression by wild-type p53. *Proc Natl Acad Sci USA* **93**: 2442–2447

Forstermann U and Kleinert H (1995) Nitric oxide synthase: expression and expressional control of the three isoforms. *Naunyn Schmiedebergs Arch Pharmacol* **352**: 351–364

Forsythe JA, Jiang BH, Iyer NV, Agani F, Leung SW, Koos RD and Semenza GL (1996) Activation of vascular endothelial growth factor gene transcription by hypoxia-inducible factor 1. *Mol Cell Biol* **16**: 4604–4613

Greenblatt MS, Bennett WP, Hollstein M and Harris CC (1994) Mutations in the p53 tumour suppressor gene: clues to cancer etiology and molecular pathogenesis. *Cancer Res* **54**: 4855–4878

Guo FH, De Raeve HR, Rice TW, Stuehr DJ, Thunnissen FB and Erzurum SC (1995) Continuous nitric oxide synthesis by inducible nitric oxide synthase in normal human airway epithelium in vivo. *Proc Natl Acad Sci USA* **92**: 7809–7813

Hanahan D and Folkman J (1996) Patterns and emerging mechanisms of the angiogenic switch during tumorigenesis. *Cell* **86**: 353–364

Harris AL (1997) Antiangiogenesis for cancer therapy. *Lancet* **349**: 13–15

Jenkins DC, Charles IG, Thomsen LL, Moss DW, Holmes LS, Baylis SA, Rhodes P, Westmore K, Emson PC and Moncada S (1995) Roles of nitric oxide in tumour growth. *Proc Natl Acad Sci USA* **92**: 4392–4396

Kang S-M, Maeda K, Onoda N, Chung Y-S, Nakata B, Nishiguchi Y and Sowa M (1997) Combined analysis of p53 and vascular endothelial growth factor expression in colorectal carcinoma for determination of tumour vascularity and liver metastasis. *Int J Cancer* **74**: 502–507

Kieser A, Weich HA, Brandner G, Marme D and Kolch W (1994) p53 potentiates protein kinase C induction of vascular endothelial growth factor expression. *Oncogene* **9**: 963–969

Kim KJ, Li B, Winer J, Armanini M, Gillett N, Phillips HS and Ferrara N (1993) Inhibition of vascular endothelial growth factor-induced angiogenesis suppresses tumour growth in vivo. *Nature* **362**: 841–844

Kobzik L, Bredt DS, Lowenstein CJ, Drazen J, Gaston B, Sugarbaker D and Stamler JS (1993) Nitric oxide synthase in human and rat lung: immunocytochemical and histochemical localization. *Am J Respir Cell Mol Biol* **9**: 371–377

Larcher F, Robles AI, Duran H, Murillas R, Quintanilla M, Cano A, Conti CJ and Jorcano JL (1996) Up-regulation of vascular endothelial growth factor/vascular permeability factor in mouse skin carcinogenesis correlates with malignant progression state and activated H-ras expression levels. *Cancer Res* **56**: 5391–5396

Lehman TA, Bennett WP, Metcalf RA, Reddel R, Welsh JA, Ecker J, Modali RV, Ullrich S, Romano JW, Appella E, Testa JR, Gerwin BI and Harris CC (1991) p53 mutations, ras mutations and p53-heat shock 70 protein complexes in human lung cell lines. *Cancer Res* **51**: 4090–4096

Maeda H, Noguchi Y, Sato K and Akaike T (1994) Enhanced vascular permeability in solid tumour is mediated by nitric oxide and inhibited by both new nitric oxide scavenger and nitric oxide synthase inhibitor. *Jpn J Cancer Res* **85**: 331–334

 © Cancer Research Campaign 1998

Mattern J, Koomagi R and Volm M (1995) Vascular endothelial growth factor expression and angiogenesis in non-small cell lung carcinomas. *Int J Cancer* **6**: 1059–1062

Mazure NM, Chen EY, Yeh P, Laderoute KR and Giaccia, AJ (1996) Oncogenic transformation and hypoxia synergistically act to modulate vascular endothelial growth factor expression. *Cancer Res* **56**: 3436–3440

Melillo G, Musso T, Sica A, Taylor LS, Cox GW and Varesio L (1995) A hypoxia-responsive element mediates a novel pathway of activation of the inducible nitric oxide synthase promoter. *J Exp Med* **182**: 1683–1693

Millauer B, Shawver LK, Plate KH, Risau W and Ullrich A (1994) Glioblastoma growth inhibited in vivo by a dominant-negative Flk-1 mutant. *Nature* **367**: 576–579

Mukhopadhyay D, Tsiokas L and Sukhatme VP (1995) Wild-type p53 and v-Src exert opposing influences on human vascular endothelial growth factor gene expression. *Cancer Res* **55**: 6161–6165

Plate KH, Breier G, Weich HA, Mennel HD and Risau W (1994) Vascular endothelial growth factor and glioma angiogenesis: coordinate induction of VEGF receptors, distribution of VEGF protein and possible in vivo regulatory mechanisms. *Int J Cancer* **59**: 520–529

Rak J, Mitsuhashi Y, Bayko L, Filmus J, Shirasawa S, Sasazuki T and Kerbel RS (1995) Mutant ras oncogenes upregulate VEGF/VPF expression: implications for induction and inhibition of tumour angiogenesis. *Cancer Res* **55**: 4575–4580

Rodenhuis S and Slebos RJ (1992) Clinical significance of ras oncogene activation and smoking in adenocarcinoma of human lung. *Cancer Res* **52**(suppl. 9): 2665s–2669s

Salter M, Knowles RG and Moncada S (1991) Widespread tissue distribution, species distribution and changes in activity of Ca(2+)-dependent and Ca(2+)-independent nitric oxide synthases. *FEBS Lett* **291**: 145–149

Shweiki D, Itin A, Soffer D and Keshet E (1992) Vascular endothelial growth factor induced by hypoxia may mediate hypoxia-initiated angiogenesis. *Nature* **359**: 843–845

Takahashi T, Suzuki H, Hida T, Sekido Y, Ariyoshi Y and Ueda R (1991) The p53 gene is very frequently mutated in small-cell lung cancer with a distinct nucleotide substitution pattern. *Oncogene* **6**: 1775–1778

Takahashi Y, Kitadai Y, Bucana, CD, Cleary KR and Ellis LM (1995) Expression of vascular endothelial growth factor and its receptor, KDR, correlates with vascularity, metastasis, and proliferation of human colon cancer. *Cancer Res* **55**: 3964–3968

Thomsen LL, Lawton FG, Knowles RG, Beesley JE, Riveros-Moreno V and Moncada S (1994) Nitric oxide synthase activity in human gynecological cancer. *Cancer Res* **54**: 1352–1354

Thomsen LL, Miles DW, Happerfield L, Bobrow LG, Knowles RG and Moncada S (1995) Nitric oxide synthase activity in human breast cancer. *Br J Cancer* **72**: 41–44

Thomsen LL, Scott JMJ, Topley P, Knowles RG, Keerie AJ and Frend AJ (1997) Selective inhibition of inducible nitric oxide synthase inhibits tumour growth in vivo: studies with 1400W, a novel inhibitor. *Cancer Res* **57**: 3300–3304

Tozer GM, Prise VE and Chaplin DJ (1997) Inhibition of nitric oxide synthase induces a selective reduction in tumour blood flow that is reversible with L-arginine. *Cancer Res* **57**: 948–955

Ziche M, Morbidelli L, Choudhuri R, Zhang HT, Donnini S, Granger HJ and Bicknell R (1997) Nitric oxide synthase lies downstream from vascular endothelial growth factor-induced but not basic fibroblast growth factor-induced angiogenesis. *J Clin Invest* **99**: 2625–2634

© Cancer Research Campaign 1998